# Losing maleness: Somatic Y chromosome loss at every stage of a man's life

**DOI:** 10.1096/fba.2019-00006

**Published:** 2019-04-02

**Authors:** Mami Miyado, Maki Fukami

**Affiliations:** ^1^ Department of Molecular Endocrinology National Research Institute for Child Health and Development Tokyo Japan

**Keywords:** infertility, karyotype, somatic mosaicism, Turner syndrome, Y chromosome

## Abstract

Mosaic loss of Y chromosome (LOY) is assumed to be among the most common acquired genetic variations in elderly people. Recent studies have linked aging‐related mosaic LOY to the risk of Alzheimer's disease, cancer, and early death. Here, we propose that mosaic LOY can present in men at any age. Mosaic LOY appears to be associated with disorders of sex development and Turner syndrome at birth, short stature from childhood, and spermatogenic failure at reproductive age, in addition to shortened survival after 60 years of age.

**Abbreviations**
DSD: disorders of sex development
LOY: loss of Y chromosome

Although the human Y chromosome harbors more than 70 protein‐coding genes, none of them are essential for the survival of somatic cells.^1^ Therefore, a 46,XY cell can stay alive in the body even when its Y chromosome is lost during mitosis. Moreover, the Y chromosome appears particularly prone to mitotic loss, because of its small size and the high frequency of palindromes that induce formation of dicentric chromosomes.^1^, ^2^ Consequently, men over 60 years of age frequently exhibit mosaic loss of Y chromosome (LOY) in peripheral blood samples.^1^, ^3^, ^4^ LOY is assumed to be among the most common acquired genetic variations in elderly people.^1^ Accumulating evidence suggest that aging‐related mosaic LOY is associated with early death, as well as the risk of Alzheimer's disease, autoimmune thyroiditis, and cancers.^3^, ^4^, ^5^ Recently, Kimura et al^6^ analyzed cross‐sectional data of the Y chromosome/X chromosome ratio in blood samples of healthy men between 20 and 70 years of age, and detected a constant decrease in the ratio with age. This suggests that the postnatal occurrence of LOY is not restricted to men over 60 years of age. To date, however, the biological significance of mosaic LOY in children and young men has not been discussed.

Herein, we propose that mosaic LOY can present in men at any age and is associated with a wide range of disorders (Figure 1). This notion is based on the re‐evaluation of the results of previous studies from the viewpoint of LOY.

**Figure 1:**
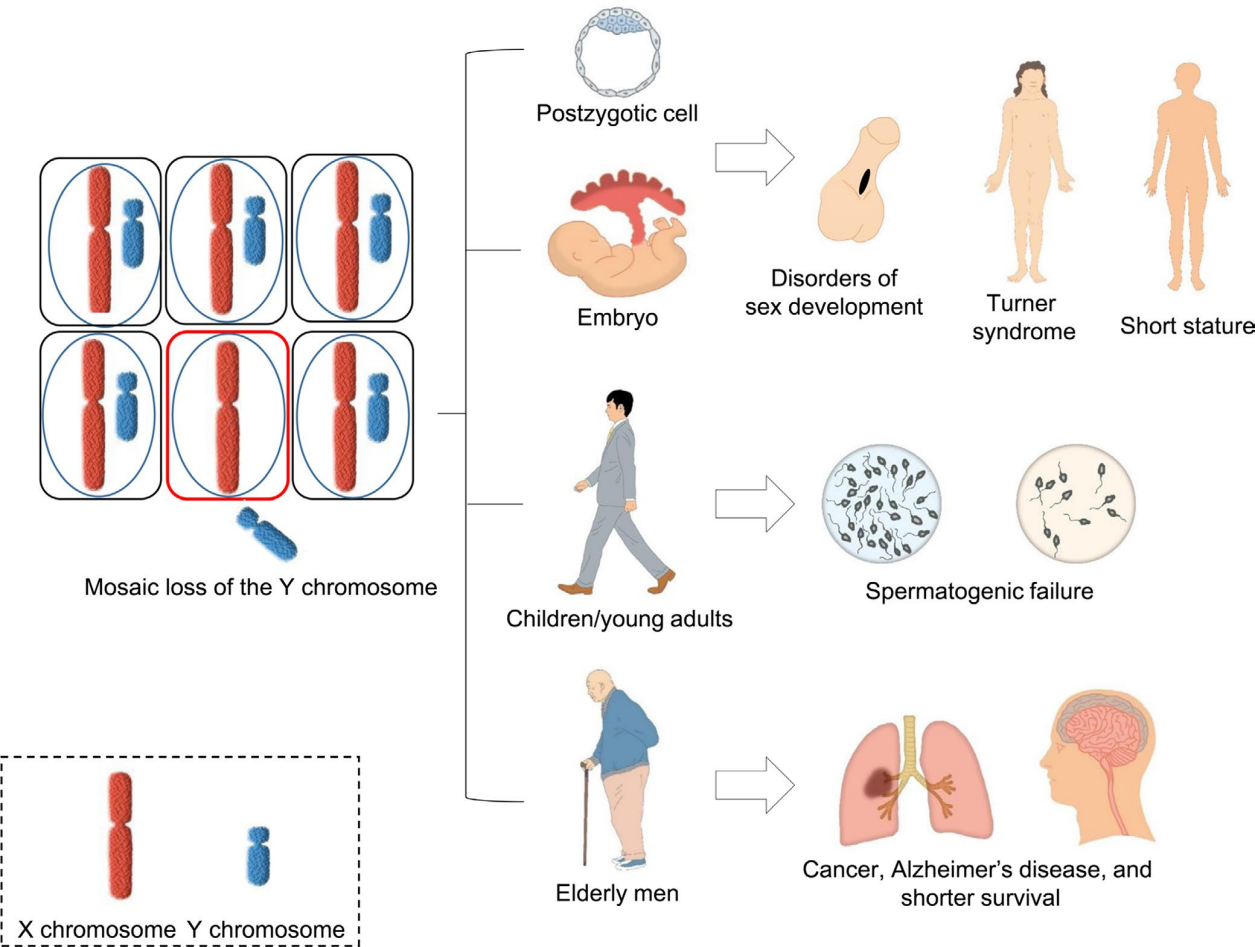
Outcomes of Y chromosome loss. Mosaic loss of Y chromosome (LOY) can present in postzygotic cells, developing embryos, children/young adults, and elderly men. The phenotypic consequences of mosaic LOY are highly variable depending on the age of the affected individuals

First, if the Y chromosome is lost in an early‐stage embryo, the embryo should have a mosaic 45,X/46,XY karyotype. This karyotype has been reported in several neonates with mixed gonadal dysgenesis, a type of disorders of sex development (DSD) characterized by incomplete masculinization of external genitalia.^7^, ^8^ These data suggest that mosaic LOY during embryogenesis constitutes one of the genetic causes of DSD. Recently, we identified a boy with hypospadias and a 45,X/46,X,idic(Y)/46,XY karyotype.^2^ This case provided evidence that dicentric Y chromosomes can be formed in somatic cells of 46,XY embryos, subsequently leading to mosaic LOY and DSD.^2^

Second, mosaic LOY during early embryogenesis may also be associated with Turner syndrome, when the 45,X cell lineage becomes predominant in the body. Turner syndrome is a relatively common condition characterized by a normal female phenotype combined with short stature and ovarian dysfunction. Although the 45,X/46,XY karyotype have been detected only in a small percentage of Turner syndrome patients, 46,XY cells may be hidden in patients with an apparent 45,X karyotype. Indeed, the current understanding is that most, if not all, Turner syndrome cases with a 45,X karyotype have cryptic mosaicism.^9^ Notably, patients with Turner syndrome and a 45,X karyotype are more prone to retain maternally derived X chromosomes than to conserve paternally derived ones (expected ratio of 2/3 vs observed ratio of approximately 3/4).^9^ These findings suggest that a considerable proportion of Turner syndrome cases is ascribable to LOY during early development and subsequent clonal expansion of the 45,X cell lineage.

Third, mosaic LOY during embryogenesis appears to be related to short stature in boys. Thus far, the 45,X/46,XY karyotype was identified in a number of boys with short stature and a normal male phenotype.^10^ In these individuals, the 45,X cell lineage may be predominant in skeletal tissues, but not in other tissues. Growth disadvantage of these individuals is ascribable to the mosaic loss of *SHOX* and other growth genes on the Y chromosome and the gross chromosomal imbalance due to X monosomy.^11^

Lastly, considering that the Y chromosome harbors a large number of spermatogenic genes,^1^ mosaic LOY in the gonads of children and young adults may be associated with defective spermatogenesis. Notably, Shin et al^12^ performed conventional karyotyping for 1,354 azoospermia patients and found that, among the various chromosomal abnormalities in 327 patients, the 45,X/46,XY karyotype was shared by eight individuals. Similarly, Yatsenko et al^13^ identified the same mosaic karyotype in one of 629 men with spermatogenic failure. Although Shin et al and Yatsenko et al did not link their findings to LOY, their results imply that mosaic LOY occurs in children and/or in young men and accounts for a small proportion of the etiology of spermatogenic failure. Alternatively, the 45,X/46,XY karyotype of azoospermia patients may reflect cellular mosaicism of the embryos, instead of postnatal LOY. Thus far, there is no evidence for the occurrence of LOY during childhood; although the tendency of aging‐related LOY was observed in men under 60 years of age, the percentage of LOY‐positive cells in these individuals were extremely low.^6^

Altogether, these findings imply that mosaic LOY can present in men at any age and is associated with DSD and Turner syndrome at birth, short stature from childhood, spermatogenic failure at reproductive age, and shortened survival and higher risk of cancers and other disorders after 60 years of age. Further studies are necessary to clarify whether mosaic LOY in children and young men exclusively occurs during embryogenesis or can be acquired after birth.

## CONFLICT OF INTEREST

The authors have declared that no competing interests exist.

## AUTHOR CONTRIBUTIONS

M. Miyado and M. Fukami conceived of the hypothesis; and M. Fukami wrote the paper.
